# Impact of the Welsh Government Flying Start programme on children’s BMI and immunisation uptake

**DOI:** 10.23889/ijpds.v9i5.2776

**Published:** 2024-09-18

**Authors:** Silvia Colonna, Kathryn Helliwell

**Affiliations:** 1 Welsh Government

Flying Start (FS) is the Welsh Government’s targeted Early Years programme for families with children under 4 years of age who live in some of the most disadvantaged areas of Wales.

The programme aims to mitigate the impact of poverty which is linked to poor life outcomes in early childhood. In particular, this study aims to investigate the impact of FS programme on FS children’s health outcomes in the form of Body Mass Index (BMI) and immunisation uptake, using linking analysis. The datasets linked to the FS cohort are respectively the Child Measurement Programme (CMP) and the National Community Child Health (NCCH). CMP consists of routinely taking height and weight measures of children in reception year to monitor BMI, whereas the NCCH is used to monitor health surveillance programmes, including immunisation. This project will use linked data to investigate the prevalence of fully immunised children across different levels of engagement with FS programme (normal and under-engagement). It will also look at prevalence of different BMI thresholds (underweight, overweight, and normal weight) and differences in mean BMI scores by FS programme engagement levels. In particular, chi-square will be used to explore differences in the proportion of fully immunised children and BMI thresholds, and t-test will be used to identify between-group differences in mean BMI scores.

Results from this programme will inform of long-term health outcomes in relation to Early Years programmes. It will also help policy target resources and inform relevant interventions to support the healthy growth of children.

